# The Fragrance of the Heifer’s Breath

**DOI:** 10.3201/eid1704.AC1704

**Published:** 2011-04

**Authors:** Polyxeni Potter

**Affiliations:** Author affiliation: Centers for Disease Control and Prevention, Atlanta, Georgia, USA

**Keywords:** art science connection, emerging infectious diseases, art and medicine, Eugène-Ernest Hillemacher, Edward Jenner Vaccinating a Boy, smallpox, vaccination, historical subjects, academic painting, about the cover

**Figure:**
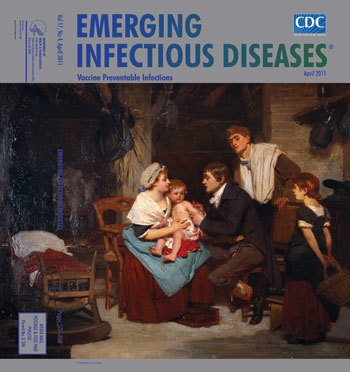
**Eugène-Ernest Hillemacher (1818–1887) *Edward Jenner Vaccinating a Boy* (1884) Oil on canvas (73.1 cm × 92.7 cm).** Copyright Wellcome Library, London

Smallpox was “the most terrible of all the ministers of death,” wrote English historian Thomas Macaulay. In the 17th century, the “small pox was always present,” unlike the plague, which visited on occasion. Before this time, the disease did not pose a serious threat to the English or other nations in Europe perhaps because, as scant available records suggest, less virulent forms of the disease were endemic. In his account of Queen Mary’s death (1694), Macaulay spoke of smallpox as indiscriminate and pernicious, attacking royalty and the poor alike, “turning the babe into a changeling at which the mother shuddered and making the eyes and cheeks of the betrothed maiden objects of horror to the lover.”

Early in the century, the disease started appearing in English poetry, and in 1616, the year of Shakespeare’s death, Ben Johnson published “An Epigram to the Smallpox”: “Envious and foule Disease, could there not be / One beauty in an Age, and free from thee?” These writings reflected growing awareness of smallpox as debilitating and deadly. By the end of the century, it had overtaken not only the plague but leprosy and the great pox, syphilis, and was spreading quickly in communities, disrupting the social fabric and causing epidemics similar to those that would sweep the globe in the 18th and 19th centuries. The general belief was that smallpox was carried from the sick on clothes and bedding, though the primary source of infection was actually particles of moisture in the patient’s breath. Infection traveled hundreds of yards by air, and the virus entered the body through the nose and mouth rather than cuts in the skin.

Malodorous and disfiguring, smallpox held none of the mystique of tuberculosis, whose wasting febrile victims would later inspire romantic literature, music, and art. On the contrary, it struck fear in the hearts of poets, from Andrew Marvel to John Donne. Those who tried their hand at elegies struggled to align this “loathsome and unlovely” condition with internal beauty. A tribute by a young John Dryden to his friend Lord Hastings, who died the day before he was to marry, illustrates the pitfalls: “Blisters with pride swell’d; which th’row’s flesh did sprout / Like Rose-buds, stuck i’th’ Lily-skin about. / Each little Pimple had a Tear in it, / to wail the fault its rising did commit: / Who, Rebel-like, with their own Lord at strife, / Thus made an Insurrection ’gainst his Life.”

Dryden’s attempt in his first published poem to liken Hastings’ pustules to rosebuds and later to gems and stars failed to transform smallpox to anything palatable. Dryden was not alone in confronting the reality of pathologic symptoms in a poem, but this disease, an everyday event, defied metaphorical interpretation and therefore transformative treatment. Moreover, it was not viewed as divine punishment for “Capital offense, / Some high, high Treason.” It remained instead, a “Fierce disease, which knows not how to spare / The young, the great, the knowing, or the Fair.”

Records from Glasgow show that in the immediate prevaccination era (1783–1800), 50% of children died before age 10, and of those deaths, 40% were due to smallpox, the leading cause of blindness in Europe. Those who survived were often badly blemished, “Beauty’s Enemy” lingering “in many a pityed face / Those hatefull pits and furrowes of its trace.”

The roots of smallpox in antiquity have been argued, as have efforts to control it. The practice of variolation―inoculation with a small amount of material from a pustule or scab of a smallpox patient—had long been known in Asia and was introduced to Europe and North America in the early 1700s. But it was not widely practiced because of the risk for disease or death to the inoculated person and the risk for creating new outbreaks.

Then Edward Jenner (1749–1823) came along. An alert physician, he observed that some in his community, mostly farmers and milkmaids frequently exposed to cowpox, did not come down with smallpox. Bucking anecdotal evidence and standard variolation, he went out on a limb. “The first experiment (14 May 1796) was made upon a lad of the name of Phipps, in whose arm a little Vaccine Virus was inserted taken from the hand of a young woman who had been accidentally infected by a cow…. On his being inoculated some months afterwards, it proved that he was secure…. As soon as I could again furnish myself with Virus from the Cow, I made an arrangement for a series of inoculations.”

Jenner wrote his observations in his *Inquiry into the Causes and Effects of the Variolae Vaccinae, A Disease Discovered in Some of the Western Counties of England, Particularly Gloucestershire, and Known by the Name of the Cow Pox*. He submitted this small but potent study for publication in the Philosophical Transactions of the Royal Society of which he was a member recognized for his contributions to the field of natural history. The manuscript was rejected, so he published it himself.

An early practitioner of One Medicine, Jenner referred to the cowpox material as “vaccine,” from the Latin *vacca* (cow), acknowledging the essential link between human and animal health. Eventually, the procedure of injecting the vaccine was generally referred to as vaccination. Louis Pasteur later insisted that all inoculations designed to protect against disease be called vaccinations in honor of Jenner and named his own discovery “rabies vaccine,” though it had no connection with cows.

*Edward Jenner Vaccinating a Boy*, on this month’s cover, commemorates vaccination against smallpox by the country doctor from Berkeley, England, who first demonstrated its effectiveness. Jenner, relying on epidemiologic observations, transformed haphazard efforts against smallpox into a public health approach to disease control and laid the foundation for eradication of this disease and the prevention of many others. This historic moment was captured by Eugène-Ernest Hillemacher, a French painter who worked in the tradition of William Bouguereau and Jean-Léon Gérôme―academic painters known for their affection toward historical subjects. A student of Léon Cogniet, Hillemacher exhibited in the Salon and became Chevalier de la Légion d’Honneur.

The artist’s rendition of Jenner’s moment in history carefully recorded it for posterity. This traditional bucolic setting shows a family in their modest country home. The back of a cow sharing the premises is visible behind the crib. The good doctor is fully engaged, comforting the young patient who is about to feel a prick. The parents lean forward trustingly. Ample light and bright colors denote optimism. This family’s future is safe from pestilential illness. But Jenner here is not tending this child alone but all children. He is pioneering public health’s crowning achievement, childhood immunization.

While the specter of smallpox did not translate into lyrical verse, the prospect of its relief resonated with poets and artists. Samuel Taylor Coleridge, whose son died after being variolated, was inspired by Jenner’s bold discovery and wrote to him. Eliciting the genius of John Milton, he rated the discovery “capable in the highest degree of being poetically treated,” by the bard’s very definition of poetry as “simple, sensuous, and impassioned.” Coleridge did not follow up, but other Romantic poets did. In 1804, Robert Bloomfield wrote in “Good Tidings or News from the Farm” about the “fragrance of the heifer’s breath.”

In the 180 years between Jenner’s earthshaking experiment and the fulfillment of his vision that “the annihilation of the Small Pox… must be the final result of this practice,” he was often berated and ridiculed, particularly for injecting animal material into humans, thus diminishing the distance between them and unclean or sick beasts. We have since learned enough about our close kinship with the animal kingdom, and the dangers involved, to look back at the origins of “vaccine” with affection. Unlike humans whose breath spread smallpox to those who “breath’d the tainted air,” Jenner’s heifer lived in the house with the farm family, sharing nothing but her fragrant breath.
